# Fibroblast growth factor-2 alleviates the capillary leakage and inflammation in sepsis

**DOI:** 10.1186/s10020-020-00221-y

**Published:** 2020-11-13

**Authors:** Xiaojun Pan, Shunyao Xu, Zhen Zhou, Fen Wang, Lingjie Mao, Hao Li, Caixia Wu, Junfeng Wang, Yueyue Huang, Dequan Li, Cong Wang, Jingye Pan

**Affiliations:** 1 Department of Intensive Care Unit, The First Affiliated Hospital of Wenzhou Medical University, Wenzhou, 325000 Zhejiang P. R. China; 2 Department of Intensive Care Unit, Hangzhou Third Hospital, Hangzhou, 310000 Zhejiang P. R. China; 3 Wenzhou Medical University, Wenzhou, 325000 Zhejiang P. R. China; 4 The Yiwu Affiliated Hospital of Wenzhou Medical University, Jinhua, 322000 Zhejiang P. R. China; 5 Department of Traumatology Medicine, The First Affiliated Hospital of Wenzhou Medical University, Wenzhou, 325000 Zhejiang P. R. China

**Keywords:** FGF2, ALI, Inflammation, Permeability

## Abstract

**Background:**

Acute lung injury (ALI), which is induced by numerous pathogenic factors, especially sepsis, can generate alveolar damage, pulmonary edema and vascular hyper-permeability ultimately leading to severe hypoxemia. Fibroblast growth factor-2 (FGF2) is an important member of the FGF family associated with endothelial cell migration and proliferation, and injury repairment. Here, we conducted this study aiming to evaluate the therapeutic effect of FGF2 in sepsis-induced ALI.

**Methods:**

Recombinant FGF2 was abdominally injected into septic mice induced by cecal ligation and puncture (CLP), and then the inflammatory factors of lung tissue, vascular permeability and lung injury-related indicators based on protein levels and gene expression were detected. In vitro, human pulmonary microvascular endothelial cells (HPMEC) and mouse peritoneal macrophages (PMs) were challenged by lipopolysaccharides (LPS) with or without FGF2 administration in different groups, and then changes in inflammation indicators and cell permeability ability were tested.

**Results:**

The results revealed that FGF2 treatment reduced inflammation response, attenuated pulmonary capillary leakage, alleviated lung injury and improved survival in septic mice. The endothelial injury and macrophages inflammation induced by LPS were inhibited by FGF2 administration via AKT/P38/NF-κB signaling pathways.

**Conclusion:**

These findings indicated a therapeutic role of FGF2 in ALI through ameliorating capillary leakage and inflammation.

## Introduction

Sepsis, featured by systemic inflammatory response syndrome (SIRS), is caused by infection and can develop into multiple-organ injury, multiple organ failure and shock (Rhodes et al. 2017). Despite the advances in healthcare, the death rates of patients with sepsis remain high at 25%–55% around the world (Fleischmann et al. 2016; Degoricija et al. 2006). The lung is extremely vulnerable to damage in various pathologies, including ischemia–reperfusion injury, post-injury multiple organ failure, influenza virus infections and sepsis, which lead to acute lung injury (ALI) (Ciesla et al. 2005; Hengst et al. 2010; Jochems et al. 2018).

Among numerous causes of ALI, overwhelming inflammation, overproduction of reactive oxygen species (ROS), endotoxin release into the circulation and capillary leakage are the main contributors (Chen et al. 2014; Wheeler and Bernard 2007). It is known that the barrier function of the pulmonary capillary endothelial is almost uniformly disrupted in septic ALI, which contributes to adverse outcomes (Birukov et al. 2013). And it is likely that this endothelium disfunction also results in a systemic cytokine storm, which causes inflammatory cell infiltration and further promotes inflammation response (Bhattacharya and Matthay 2013). The pathological process of ALI incorporates the alveolar–capillary membrane and the adhesion, activation of inflammatory cells, which deteriorate the damage to organisms (Wang et al. 2002; Aggarwal et al. 2014). Base on this, treating sepsis by alleviating pulmonary dysfunction is one of the potential therapeutic methods. Unfortunately, various mechanical ventilation and drug therapies have failed to distinctly improve sepsis survival until now (Matthay et al. 2019). Therefore, therapeutic opinions that can effectively remedy and decrease the mortality of sepsis must be urgently established.

Fibroblast growth factor-2 (FGF2), also known as basic fibroblast growth factor, is the first discovered prototypical members of the FGF family, which plays pleiotropic roles in cellular and metabolic homeostasis (Li 2019). A study shown that FGF2 stimulated migration and proliferation of endothelial cells in vivo and inhibit inflammation (Zbinden et al. 2018; Decker et al. 2016). Another reported that FGF2 cooperating with IL-17 promoted the pathogenesis of autoimmune arthritis, other reported FGF2 suppressed the neuroinflammation through the FGF2-ERK1/2 signaling, demonstrating that FGF2 inactivated ERK1/2 in the hippocampus and the microglial marked by Iba1 in CA1, CA2, CA3 and DG (Shao et al. 2017; Tang et al. 2017). To ascertain whether the FGF2 positively or negatively affected the inflammation in ALI, we examined its role in vivo and vitro.

## Materials and methods

### Reagents and antibodies

LPS (*Escherichia coli* O111:B4) and peroxidase from horseradish (HRP, P8375) were purchased from Sigma Aldrich Company. Human recombinant FGF2 (100-18B) was acquired from PeproTech. Evans Blue (0109A14) was bought from Leagene, China. Wright-Giemsa (G1020), 4% paraformaldehyde (P1110), 5% BSA (SW3015) and Triton X-100 (T8200) were procured from Solarbio Life Sciences (Beijing, China). Anti-GAPDH (SC-47724), anti-α-E-catenin (SC-9988), anti-IL-6 (SC-57315), anti-ROBO4 (SC-166872) and anti-F4/80 (SC-377009) were bought from Santa Cruz Biotechnology Inc., (Santa Cruz, CA). Anti-AKT (4691), anti-p-AKT (4060), anti-β-actin (4970), anti-P65 (9936), anti-p-P65 (9936), anti-P38 (8690), anti-p-P38 (9216), anti-IL-1β (12242), anti-TNF-α (3707) and DAPI (4083) were acquired from Cell Signaling Technology, USA. Anti-VE-cadherin (ab33168), anti-rabbit IgG H&L (FITC, ab6717), anti-Mouse IgG H&L (Cy3, ab97035) and anti-mouse IgG H&L (FITC, ab6785) were obtained from Abcam (Cambridge, MA). Anti-cyclooxygenase 2 (COX2, bs-0732R) was acquired from Bioss Inc. (Beijing, China). Anti-mouse HRP-conjugated polyclonal antibody (170–6516) and anti-rabbit HRP-conjugated polyclonal antibody (170–6515) were garnered from Bio-Rad, USA. Phenylmethanesulfonyl fluoride (PMSF) were acquired from Beyotime, China. BCA kit and Radio-Immunoprecipitation Assay (RIPA) were received from Thermo Scientific, USA. Protein phosphatase inhibitor mixture (P1260) was gained from Applygen (Beijing, China).

### Animals

Male C57BL/6 mice (8–10 weeks old, 18–25 g/body) were purchased from Shanghai SLAC Laboratory, Animal Limited Liability Company (Shanghai, China). The animals were fed in accordance with the local animal welfare policy with an ambient temperature of 23 ± 3 °C, a relative humidity of 55% ± 10%, and a 12 light/dark cycle according to the Institutional Animal Care and Use Committee’s guidelines.

### CLP and animal tests

The mice were anaesthetized and prepared for the CLP model as previously described (Giano et al. 2014). The mice were randomly divided into three groups: sham, CLP and CLP with FGF2. Simply, mice were operated with an abdominal incision, the cecum was ligated and punctured twice with a 21-gauge needle (Kindly, Shanghai, China) to gently extrude some feces. Then the cecum was put back to the abdominal cavity and the abdomen was sutured carefully. The sham group was operated in the skin and abdominal muscle rather than subjected to CLP, and the CLP with FGF2 was peritoneally injected with 0.1 mg/kg of FGF2 protein 6 h after surgery. The untreated group was given equal amounts of phosphate buffered saline (PBS). All the mice were immediately injected with 1 ml of pre-warmed saline post-operation immediately. All mice were assessed every 12 h for the following 4 days and euthanized at the moribund stage. The experimental samples were collected 24 h after CLP.

### ELISA test

The plasma was measured using the ELISA Kit of Tumor Necrosis Factor-α (TNF-α) and interleukin-6 (IL-6) (R&D Systems, Inc., USA). The laboratory procedure was manipulated according to manufacturer's instructions. Simply put, every reagent was placed in pore plate for a period of time and cleaned many times. When the color developed, the reaction liquid didn’t be poured out but added the stop solution. The result was tested by a microplate reader (Molecular Devices, Hercules, CA, USA).

### Proteome Profiler Array analyses

One mouse was selected from each group by detecting the content of various indexes in the Mouse Angiogenesis Antibody Proteome Profiler Array (R&D Systems, Inc., USA). All array data were processed in accordance with the manufacturer’s instructions. The result was examined by VilBer LouRMAT (Bio-Rad, Universal Hood II, USA).

### Histology

The three groups of mice were sacrificed, and the right lung was harvested as previously described (Xu et al. 2018). Formalin-fixed tissues were processed for 4 h, embedded in paraffin and cut into 3 mm sections. Then, the sections were stained with hematoxylin and eosin (H&E). All sections were examined and graded based on an arbitrary four-grade scale (Zhou et al. 2007). The result was assessed in terms of airway epithelial necrosis, intra-alveolar edema, hyaline membranes, hemorrhage, and the recruitment of inflammatory cells into the air space.

### Wet/dry weight ratio

The left lung was blot up of the blood on the surface of the tissue for weighing and recording. Afterward, lung was placed in a dryer at 60 °C for 72 h and then reweighed to calculate the wet/dry (W/D) weight ratio.

### Pulmonary permeability

Pulmonary permeability was assessed as previously described (Yang et al. 2017). All mice were administered with 200 µl of 5% Evans Blue through a tail vein injection at 1 h prior to execution. The mice were narcotized and immobilized on a frame to perform transcardial perfusion with normal saline until the lung paled. Then the lung was harvested, washed, weighed and immerged in 1 ml/100 mg of formamide. After incubation at 60 °C for 24 h, the optical density (OD) value of the supernatant was detected by microplate reader (Molecular Devices, Hercules, CA, USA).

### Collection of broncho-alveolar lavage fluid (BALF)

The mice were anesthetized and fixed to collect the BALF as previously described (Yang et al. 2015). The lung tissues were lavaged thrice with 1 ml of PBS and filtrated with a metal net. The total cell number and the different cell types were determined using cytological smears, and the protein level was evaluated by the BCA kit (Thermo Scientific, USA).

### Cell culture and experiments

Human pulmonary microvascular endothelial cells (HPMECs, 3000, ScienCell, USA) were cultured in endothelial cell medium (ECM, 1001, ScienCell, USA), matched with 5% fetal bovine serum (FBS) and 1% penicillin/streptomycin in a humidified incubator under 5% CO_2_ at 37 °C. The HPMECs were seeded into the culture dish for one night. The medium was replaced with fetal bovine serum (FBS) medium for 6–8 h. The HPMECs were cultured with 1 μg/ml LPS with or without 50 ng/ml of FGF2 for 0.5 h or 6 h.

Peritoneal macrophages (PMs) were isolated from 6–8 weeks old male C57BL/6 mice. Briefly, mice were intraperitoneal injected with 2 ml Starch broth (Sigma, USA), and then cold RPMI 1640 medium was used to wash the peritoneal cavity several times to isolate peritoneal exudate cells. The cells were cultured in a humidified incubator for 2 h and the adhered cells were collected as macrophages for further experiments. PMs were cultured with 1 μg/ml LPS with or without 50 ng/ml of FGF2 for 0.5 h or 6 h.

### Endothelial tube formation assay

Briefly, the HPMECs (10 × 104 cells/well) were seeded into 24-well plates, which were covered with 120 μl of polymerized matrigel (Corning, 354234). Then the HPMECs were treated with 1 μg/ml LPS with or without 50 ng/ml of FGF2. After incubation for 3.5 h at 37 °C under 5% CO2, images were captured with an Olympus CKX41 light microscope. Each experiment was performed at least three times.

### HPMECs permeability assay

The HPMECs (5 × 10^4^ /well) were seeded in the upper chamber of 24-well Transwell plate (0.4 μm pore size; Corning Incorporated, NY) cultured at 37 °C under 5% CO_2_ until a monolayer was formed. After LPS stimulation, the HRP determined the permeability of the HPMECs permeability by measuring the concentration of the HRP in the lower chamber. The HRP was diluted with the cell culture medium, and 200 μl of HRP solution was added to the apical compartment at a concentration of 50 ng/ml. After the addition of HRP for 15 min, 10 μl of fluid was collected from the basolateral compartment of each filter. This fluid was added to 96-well plates together with 150 μl substrate and incubated at 37 ℃ for 10 min. Then, sulfuric acid was added to terminate the reaction. The OD value was measured with a microplate reader.

### Immunofluorescence

The 4 μm thickness lung sections were dewaxed, rehydrated, and antigens retrieved by sodium citrate buffer (0.01 M, pH 6.0) for 10 min. The pre-treated lung sections were blocked with 5% BSA for 30 min and incubated with the primary antibodies overnight at 4 °C. The sections were then incubated with Alexa Fluor labeled secondary antibody for 1 h at room temperature and nuclei were stained with DAPI for 5 min.

HPMECs were fixed, permeabilized, blocked, and incubated with primary antibodies overnight at 4 °C. The slides were then incubated with Alexa Fluor labeled secondary antibody for 1 h at room temperature and DAPI staining for 5 min. Images were obtained with a laser confocal microscope (Leica, Germany).

### Western blotting

The samples were dissociated by RIPA with 1% PMSF and 1% protein phosphatase inhibitor mixture, and the protein concentrations were detected using a BCA kit. The equivalent protein was separated by electrophoresis, transferred, blocked and incubated with the first antibodies overnight at 4 °C. The polyvinylidene difluoride (PVDF) membranes (EDM Millipore, Billerica, MA) were incubated with homologous species secondary antibody. After incubation, the membranes were detected by using ECL Chemiluminescent Reagent (Advansta, USA).

### Quantitative real-time RT-PCR

Trizol reagent (Invitrogen, CA) was added to every sample and the RNA was extracted using the Trizol reagent (Invitrogen, CA). The cDNA synthesis was performed using the GoScript Reverse Transcription System kit (Promega, USA) according to the manufacturer’s protocols. Quantitative real-time polymerase chain reaction (qRT-PCR) was performed with LightCycler (Roche Diagnostics, Mannheim, Germany) and SYBR Green (Roche Diagnostics, Mannheim, Germany). The primer sequences are listed in Tables 1 and 2.

**Table 1:** Mouse primer sequences for the real-time PCR

Gene	Forward primer	Reverse primer
GAPDH	GCACAGTCAAGGCCGAGAAT	GCCTTCTCCATGGTGGTGAA
IL-1β	TGCCACCTTTTGACAGTGATG	TTCTTGTGACCCTGAGCGAC
IL-6	GCCTTCTTGGGACTGATGCT	TGTGACTCCAGCTTATCTCTTGG
IL-10	TAAGGCTGGCCACACTTGAG	GTTTTCAGGGATGAAGCGGC
TNF-α	ACCCTCACACTCACAAACCA	ACCCTGAGCCATAATCCCCT
CXCL1	GGATGCCACAGGATTCCATA	GTGCCATCAGAGCAGTCTGT
CXCL10	CCAAGTGCTGCCGCTCATTTTC	TCCCTATGGCCCTCATTCTCA
MCP-1	TAAAAACCTGGATCGGAACCAAA	GCATTAGCTTCAGATTTACGGGT
TLR4	TGCAAGGAGGTTCAGTGCTC	AGTGAATGAGCCCCAGCAAA
ICAM1	TTCTCATGCCGCACAGAACT	TCCTGGCCTCGGAGACATTA

**Table 2:** Human primer sequences for the real-time PCR

Gene	Forward primer	Reverse primer
GAPDH	GGAGTCCACTGGCGTCTTC	GGTTCACACCCATGACGAAC
IL-1β	ATGATGGCTTATTACAGTGGCAA	GTCGGAGATTCGTAGCTGGA
IL-6	ACCCCCAATAAATATAGGACTGGA	AGAAGGCAACTGGACCGAAG
IL-10	TACGGCGCTGTCATCGATTT	TAGAGTCGCCACCCTGATGT
TNF-α	TGGGATCATTGCCCTGTGAG	GGTGTCTGAAGGAGGGGGTA
CXCL1	GCAGGGAATTCACCCCAAGA	GGTAGCCCTTGTTTCCCCC
CXCL10	AGCAGAGGAACCTCCAGTCT	ATGCAGGTACAGCGTACAGT
MCP-1	TTCCCCTAGCTTTCCCCAGA	TCCCAGGGGTAGAACTGTGG

### Statistical analyses

Statistical analyses were conducted using SPSS software version 20.0 (SPSS Inc., IL, USA). The categorized variables between groups were examined using one-way ANOVA or Student’s t test. All data are presented as means ± SD (standard deviation). P < 0.05 was considered statistically significant.

## Results

### FGF2 reduces the inflammatory response and improves the survival in septic mice

**Fig. 1:**
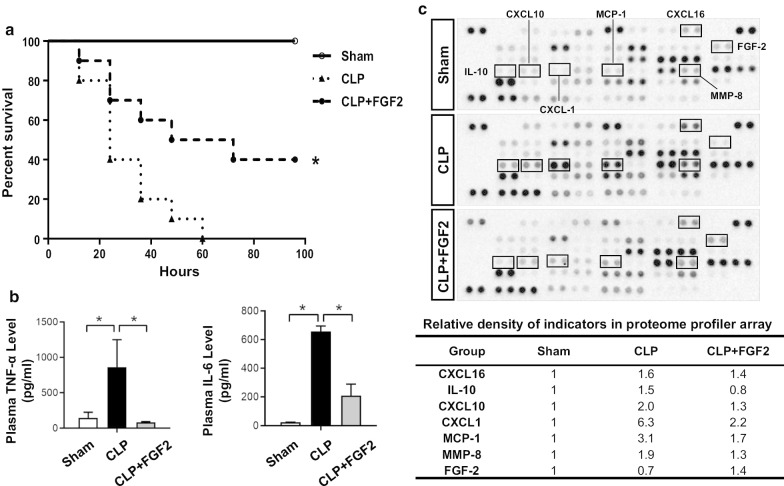
FGF2 relieves the inflammatory response and improves survival in CLP-induced sepsis. **a** The survival rate for the experimental mice. The 8–10 weeks old C57BL/6 mice were subjected to CLP, intraperitoneal injected FGF2 (0.1 mg/kg) after 6 h, and prepared as sham group as well (n = 10/group; Kaplan–Meier survival analysis). **b** The plasma levels of TNF-α and IL-6 were measured by ELISA kits at 24 h after CLP (n ≥ 5 per group). **c** Plasma analyzed by angiogenesis antibody proteome profiler array and some indexes changed obviously were quantitatively analyzed. *P < 0.05

To verify whether FGF2 exerted a protective effect against sepsis, the mice were intraperitoneally injected with FGF2 at 6 h post-operation. Our results showed that FGF2 administration reduced the mortality in septic mice (Fig. 1a), which followed by a down-regulation of plasma inflammatory factors (TNF-α and IL-6) expression (Fig. 1b). In addition, the proteome profiler array was conducted to verify that the other elevated cytokines, such as CXCL1, CXCL10, CXCL16, MCP1, MMP8, and IL-10 in the CLP group were decreased by FGF2 treatment (Fig. 1c).

### FGF2 protects the pulmonary function and mitigates the injury in septic ALI

**Fig. 2:**
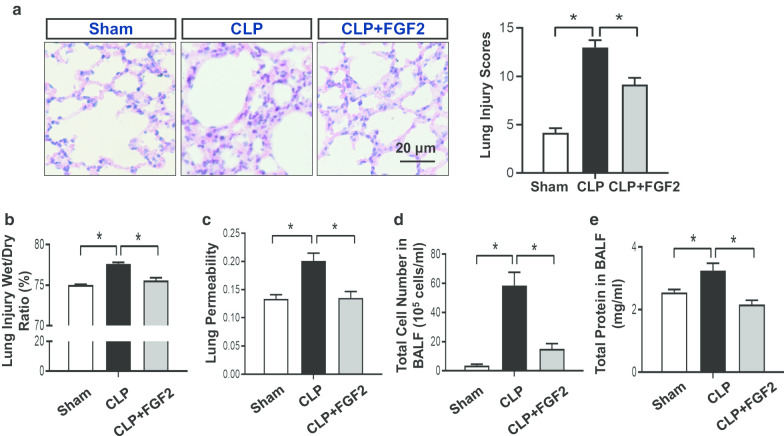
FGF2 controls the pathology and hyperosmotic state in septic lungs. **a** The different groups of lung tissue sections were stained by hematoxylin–eosin (H&E) and analyzed by the lung injury scores. **b** The lung levels of wet/dry weight ratio. The formula: ratio = (wet-dry)/wet (n ≥ 5 per group). **c** The measurement of Evans Blue permeated into mesenchyme of lung reflecting the permeability of vessel was detected by microplate reader (n ≥ 5 per group). **d** Total cell numbers in BALF were calculated with hemacytometer (n ≥ 5 per group). **e** The protein level of BALF was evaluated by the BCA kit (n ≥ 5 per group). Data are represented as mean ± SEM. *P < 0.05

The lung is extremely impaired by acute injury, and ALI is one of the complications of severe sepsis. To investigate whether FGF2 reduced the pulmonary injury caused by CLP, the lung sections were subjected to H&E staining for histological examination. Briefly, the pulmonary alveoli were not of uniform size and the alveolar septum was thicker in the CLP group than in the sham group. In comparison, the FGF2-treated group was improved (Fig. 2a). The mean lung injury score in the CLP group was 13 ± 1.79, which was much higher than that (4.17 ± 1.17) of the sham group and that (9.17 ± 1.72) of the FGF2 group (Fig. 2a). Likewise, the lung wet/dry ratio was reduced in FGF2 group compared with CLP group (Fig. 2b). To measure the capillary function, we observed its permeability using Evans Blue dye assay and found that the hyper-permeability in the CLP group was reversed by FGF2. (Fig. 2c). Afterwards, we measured the cells and concentration of protein in the BALF. We found that the count of total cells and protein in the BALF were increased in CLP group, while decreased by FGF2 administration (Fig. 2c, d).

### FGF2 attenuates the pulmonary inflammation in sepsis

**Fig. 3:**
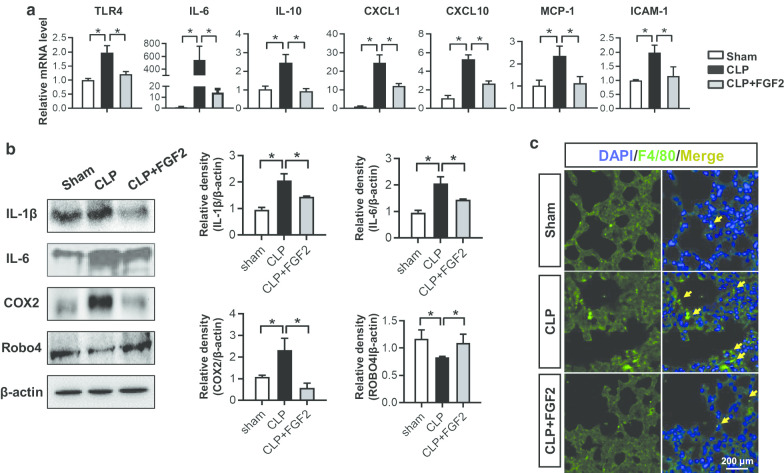
FGF2 reduced the inflammatory response in ALI. **a** Relative lung gene expressions of TLR4, IL-6, IL-10, CXCL1, CXCL10, MCP-1 and ICAM-1 were measured by quantitative real-time RT-PCR (n ≥ 5 per group, relative to GAPDH). **b** The IL-1β, IL-6, COX2, Robo4 and β-actin protein levels in the lung tissues 24 h after the surgery by western blot and the quantification of protein expressions were shown (n ≥ 3 per group). The data are represented as mean ± SEM. **c** The F4/80 on behalf of macrophage of lung tissue sections was stained by immunofluorescence. The sections are all shown at ×400 magnification. *P < 0.05

Moreover, the qRT-PCR analyses for lung tissues indicated that the mRNA expressions of TLR4, IL-6, IL-10, CXCL1, CXCL10, MCP-1 and intercellular cell adhesion molecule-1 (ICAM-1) in CLP group were higher than that in sham and FGF2-treated group (Fig. 3a). Besides, IL-6 and other inflammatory mediators, which included the TNF-α, IL-1β and COX2 protein expression, were elevated in CLP mice, while declined under FGF2 treatment (Fig. 3b). ROBO4, which is related to endothelial permeability, is one of the most important functional proteins of vessel (London et al. 2010). Our findings revealed that the FGF2 effectively improved the ROBO4 expression which was decreased in CLP lungs (Fig. 3b). As the adhesion of macrophages was related to the chemokines, such that macrophages sticking occurred in the injured lungs. We labeled F4/80 in lung tissues and found that the CLP group had more macrophages than the control group and FGF2 group (Fig. 3c).

### FGF2 alleviates the inflammation caused by LPS in HPMECs via P38/AKT/NF-κB signaling

**Fig. 4:**
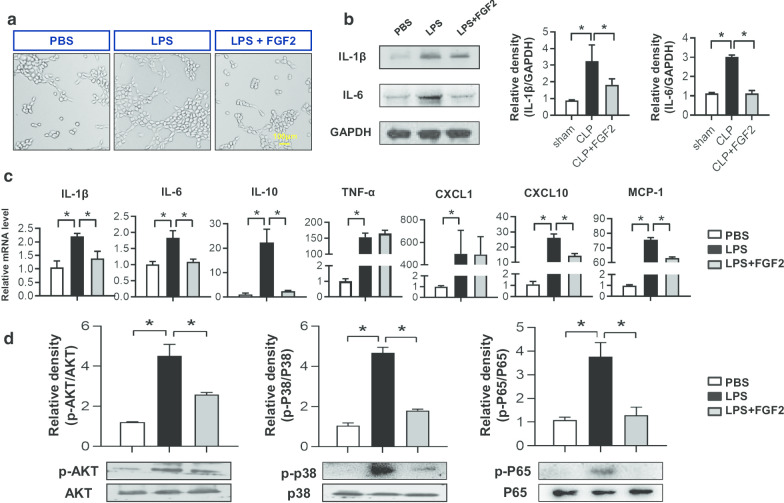
FGF2 relieves lung endothelial cells inflammation induced by LPS. **a** Tube formation assay. HPMECs were seeded into 24-well plates covered with Matrigel antecedently in different medium and observed 3.5 h after LPS (1 μg/ml) with/without FGF2 (50 ng/ml) stimulation. **b** The inflammatory cytokine IL-1β and IL-6 protein levels of HPMECs 6 h after LPS were assessed by western blot and relative densities were analyzed (n ≥ 3 per group, GAPDH as a internal control)., while the protein levels of p-p38, p38, p-AKT, AKT, p-ERK and ERK was affected 0.5 h. **c** Relative gene expressions of inflammatory factors IL-1β, IL-6, IL-10, TNF-α, CXCL1, CXCL10 and MCP-1 was measured by quantitative real-time RT-PCR (n ≥ 4 per group). Data are represented as mean ± SEM. **d** The protein expressions of p-P38, P38, p-AKT, AKT, p-P65 and P65 in HPMECs which were stimulated by LPS for 0.5 h were measured by western blot analysis and relative densities were quantified respectively (n ≥ 3 per group). * P < 0.05

Basing on the different mensuration time points and concentration, we chose the most suitable condition as follows: 50 ng/ml of FGF2 concentration for 6 h but 30 min to phosphorylate protein. To protect against exogenous damage, angiopoiesis would occur in the phase of new tissue formation. The tube formation assay revealed that the HPMECs stimulated by LPS had a higher tube formation capacity than that of the PBS and FGF2 managed groups (Fig. 4a). According to the data in vivo, the proteins of IL-1β and IL-6 were tested by Western blot analysis, which showed that both indexes of the LPS group were more expressed than the control group, while decreased under FGF2 treatment (Fig. 4b). The inflammatory mediators mRNA levels of IL-1β, IL-6, IL-10, TNF-α, CXCL1, CXCL10 and MCP-1 in LPS group were elevated compared to those of the PBS control group, while reduced in FGF2 group except for TNF-α and CXCL1 levels (Fig. 4c). In addition, we checked the relative pathways in HPMECs and found that P38 activation by phosphorylated P38 (p-P38), AKT activation by phosphorylated AKT (p-AKT) and NF-κB activation by phosphorylated P65 (p-P65) under LPS challenge were inhibited by FGF2 administration (Fig. 4d). Therefore, these results demonstrated that FGF2 controlled the inflammatory mediators through P38/AKT/NF-κB signaling pathways in HPMECs.

### FGF2 protects HPMECs from the LPS-induced injury.

**Fig. 5:**
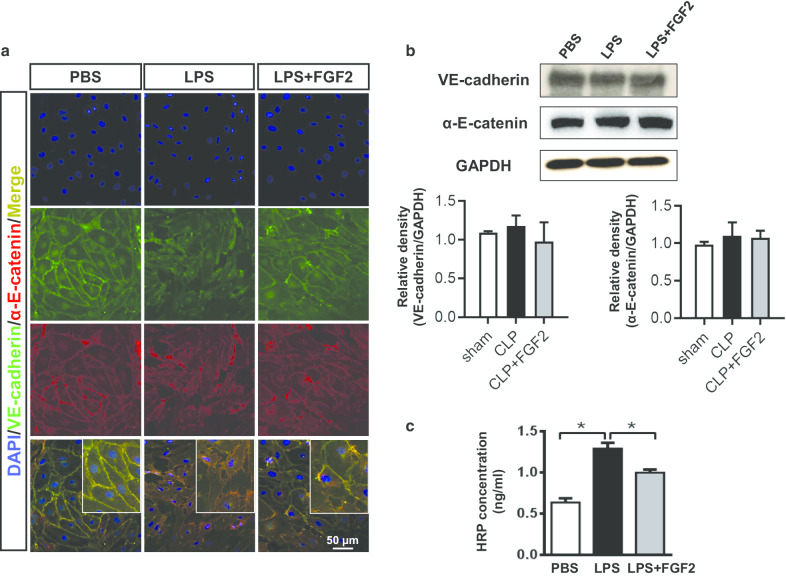
FGF2 protects the HPMECs permeability from LPS by stabilizing VE-cadherin and α-E-catenin. **a** The VE-cadherin and α-E-catenin was stained by cytofluorometry. The pictures are all shown at ×400 magnification. **b** The protein levels of intercellular connectivity VE-cadherin and α-E-catenin in HPMECs 6 h after LPS were evaluated by western blot and the relative densities were analyzed (n ≥ 3 per group, GAPDH was used as internal control). **c** HPMECs were seeded into the upper chamber of Transwell (0.4 μm) with different treatments for measuring the HRP condition (n ≥ 3 per group). Data are represented as mean ± SEM. * P < 0.05

To verify the contribution of FGF2 in improving the capillary leakage, the expression of VE-cadherin and α-E-catenin proteins, as representatives of the interlinkage in endothelial cells, were subjected to double-color immunofluorescence when the cells formed a monolayer. In the diagram, the HPMECs junction indicated by VE-cadherin and α-E-catenin immunofluorescence staining suffered from a disruption caused by LPS, although the FGF2 group was not as persecuted as the LPS group (Fig. 5a). However, the immunoblotting results showed that neither LPS nor FGF2 had impacts on VE-cadherin and α-E-catenin protein level in cell lysate (Fig. 5b). The concentration of HRP passed into the lower chamber reflected the integrity of the HPMECs. We found that the worsen HRP leakage induced by LPS stimulation was partly recovered with FGF2 administration (Fig. 5c).

### FGF2 reduced the LPS-stimulated inflammatory factors production through P38/AKT/NF-κB signaling

**Fig. 6:**
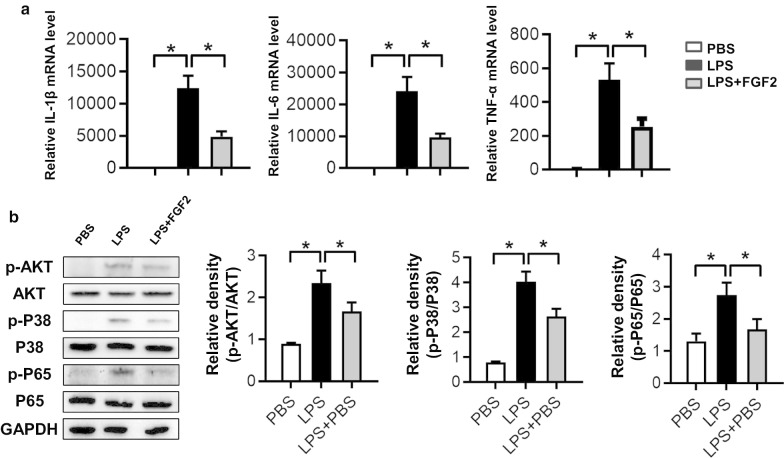
The LPS-induced proinflammatory mediators in PMs were controlled by FGF administration. **a** The mRNA expressions of IL-1β, IL-6 and TNF-α in PMs stimulated by LPS (1 μg/ml) for 6 h with or without FGF2 (50 ng/ml) administration (n ≥ 3 per group). **b** The protein levels of p-P38, P38, p-AKT, AKT, p-P65 and P65 in PMs stimulated by LPS for 0.5 h were measured by western blot and relative densities were quantified (n ≥ 3 per group, β-actin was used as a internal control). Data are represented as mean ± SEM. * P < 0.05

As FGF2 can effectively inhibit the pulmonary infiltration of macrophages in septic mice (Fig. 3c), we extracted peritoneal macrophages to check whether the proinflammatory condition in macrophages induced by LPS was controlled by FGF2 administration. Our results showed that the increasing mRNA levels of IL-1β, IL-6 and TNF-α caused by LPS stimulation were down-regulated by FGF2 administration (Fig. 6a). Unsurprisingly, the activation of P38/AKT/NF-κB pathways in macrophages by LPS challenge were restrained by FGF2 treatment (Fig. 6b). These findings revealed that FGF2 reduced the proinflammatory factors in macrophages via P38/AKT/NF-κB pathways.

## Discussion

Sepsis is considered as a systemic inflammatory response to infection, When pathogen-associated molecular patterns (PAMPs) on the surface of pathogens are recognized by related receptors on the body's immune system cells, the immune activation pathway of infection was stimulated (Boyd et al. 2014), for example by activating NF-κB signaling pathways and neutrophils cells releasing proinflammatory and anti-inflammatory mediators (Hotchkiss and Karl 2003), cytokines and complement activation (Cecconi et al. 2018). The body has an inflammatory hyperreaction to infection, and organ dysfunction caused by uncontrolled inflammation leads to death. Our research confirmed that intraperitoneal injection of FGF2 reduced the level of serum inflammatory factors, including CXCL1, CXCL10, CXCL16, MCP1, MMP8, and IL-10, which may be the possible reason for the decreased mortality of mice in the CLP.

The lung is considered to be the primary target organ damaged by sepsis, leading to ALI, and or even developing acute respiratory distress syndrome (ARDS), which is the main cause of sepsis death (Bao et al. 2010; Phua et al. 2009). The pathogenesis of ALI is a series of inflammatory mediators released by neutrophils, macrophages, endothelial cells and other cells. Inflammatory cells and mediators infiltrate into the lung, causing increased pulmonary microvascular permeability, pulmonary interstitial, alveolar edema and alveolar destruction, resulting in acute hypoxic respiratory insufficiency or respiratory failure (Herold et al. 2013). The results from both laboratory and pathological experiments in our study confirmed that after FGF2 treatment the infiltration of inflammatory factors in the lungs of CLP mice were reduced thereby reducing lung damage in mice.

As a member of the FGF family, the role of FGF2 in organ development, tissue repair, regeneration, and diseases has been widely investigated (Beenken and Mohammadi 2009). FGF2 promoted cell proliferation, activated migration and induced changes in cell shapes in many organs to leading diseases, such as breast cancer, corneal endothelial cells, cartilage regeneration and so on (Ricard et al. 2014; Song et al. 2015; Lee et al. 2018). However, whether the FGF2 has a therapeutic effect in sepsis and ALI has never been reported.

Inflammation is the main cause of endothelial barrier hyperosmolarity, which is associated with ALI and acute respiratory distress syndrome (ARDS) (Matthay et al. 2019; Barabutis et al. 2018). Inflammatory factors adhere activated immune cells to the blood vessel wall through ICAM1, synthesize chemokines to attract immune cells, endothelial cell dysfunction and necrosis that lead to increased vascular permeability (Chousterman et al. 2017), our study found that after FGF-2 treatment in mice, ICAM-1 expression decreased in lung tissue, which may reduce pulmonary capillary leakage and reduce the wet-to-dry weight ratio of mouse lungs. Even more, the proinflammatory cytokines storm breaks the barrier function of endothelial cells, endothelial injury will promote inflammation (Zbinden et al. 2018; Flemming et al. 2015), therefore, FGF2 promotes the repair of lung endothelial cells and reduces lung inflammation. ROBO4 is related to endothelial permeability, is one of the most important functional proteins of vessel, inflammatory stimulus damaged the barrier function of vessel through ROBO4-slit signaling pathway (London et al. 2010). Our data suggested that the administration of FGF2 can effectively modulate the CLP-induced ALI and also our finding also revealed that the CLP-induced decline of ROBO4 protein expression may rescued by FGF2 followed with alleviated inflammation cells infiltration in lungs.

Previous studies revealed that it is effective to inhibit Influenza H1N1 Virus Infection through regulating the expression of FGF2 by miR-194 (Wang et al. 2017). In this report, we demonstrated that FGF2 alleviated pulmonary inflammation and improved the survival in CLP mice. Moreover, we showed that FGF2 inhibited pulmonary injury and improved capillary permeability through regulating inflammatory mediators.

**Fig. 7:**
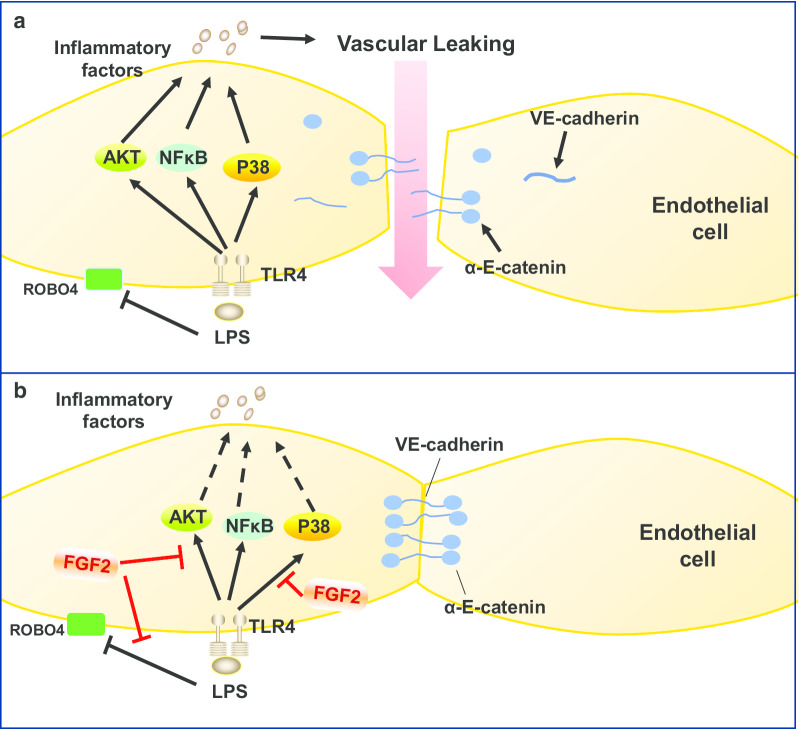
FGF2 alleviates the inflammation and vascular leakage in sepsis. FGF2 reduces vascular leakage caused by relieving inflammation through AKT/P38/NF-κB pathways. **a** LPS combined with TLR4 activates AKT/P38/NF-κB pathways to release amount of inflammatory factors leading to VE-cadherin/α-E-catenin disruption and endothelial cell barrier malfunction. **b** FGF2 enhances vascular barrier function against inflammatory factors production by inactivating AKT/P38/NF-κB pathways and stabilizing VE-cadherin/α-E-catenin at the cell surface

The endothelium is a part of the vascular system that regulates the exchange of molecules and fluids between blood circulation and body tissue compartments (Wettschureck et al. 2019). Various cytokines and chemokines contribute to endothelial cell damage and/or transient compromise of the endothelial barrier function (Harrison 2010; Teijaro et al. 2011). The proinflammatory cytokines storm breaks the barrier function of endothelial cells and the junction between VE-cadherin and α-E-catenin through ROBO4-slit signal (London et al. 2010; Yu et al. 2014; Rocheteau et al. 2015). Meanwhile, the inflammatory mediators regulatory mechanisms were highly related to mitogen-activated protein kinase (MAPK) and NF-κB signaling pathways during pathological process of sepsis. Herein, we reported that FGF2 not only markedly controlled inflammation by regulating inflammatory factors, chemotactic factors via P38/AKT/NF-κB pathways, but also protected the link of VE-cadherin and α-E-catenin proteins on the cell membrane to hold the linkage in LPS-challenged HPMECs (Fig. 7). In addition, our results also revealed that the increasing proinflammatory mediator productions induced by LPS in peritoneal macrophages were reduced by FGF2.

## Conclusion

FGF2 was beneficial in sepsis-induced ALI by alleviating inflammation and attenuating endothelial permeability via P38/AKT/NF-κB pathways, which indicated that FGF2 is a new promising treatment for sepsis.

## Data Availability

Not applicable.

## Abbreviations

ERK: Extracellular regulated protein kinases
BSA: Bovine serum Albumin
GAPDH: Glyceraldehyde-phosphate dehydrogenase
IL: Interleukin
DAPI: 4′,6-Diamidino-2-phenylindole
ELISA: Enzyme-linked Immunosorbent Assay
RPMI: 1640 Rswell park memorial institute
HRP: Peroxidase from horseradish
PMs: Peritoneal macrophages
HPMEC: Human pulmonary microvascular endothelial cells
